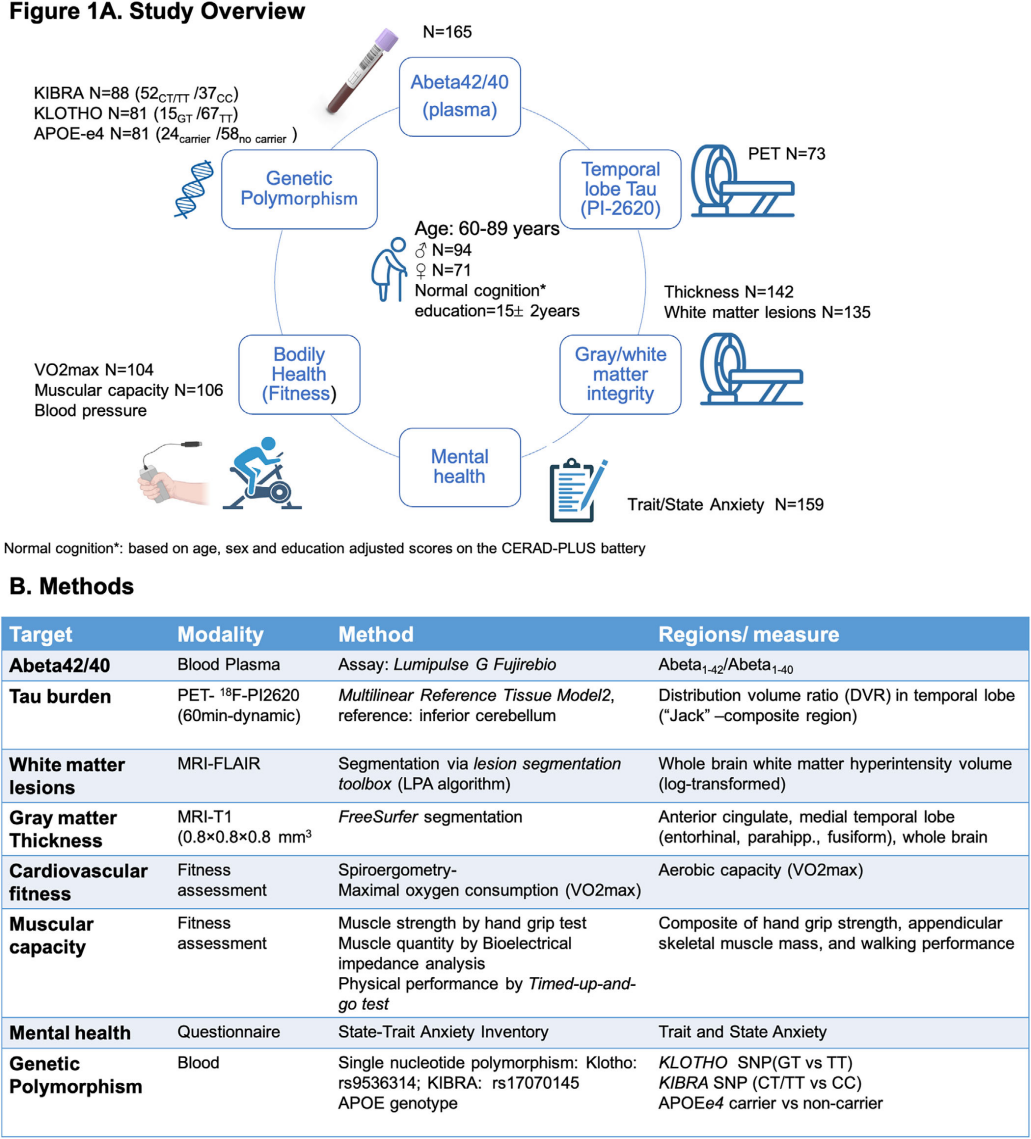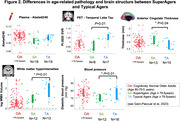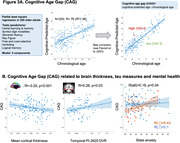# Multimodal phenotyping of successful cognitive aging

**DOI:** 10.1002/alz.093952

**Published:** 2025-01-09

**Authors:** Niklas Behrenbruch, Eóin N. Molloy, Svenja Schwarck, Beate Schumann‐Werner, Berta Garcia‐Garcia, Niklas Vockert, Barbara Morgado, Michael Rullmann, Anne Hochkeppler, Larissa Fischer, Kathrin Baldauf, Peter Schulze, Patrick Müller, Andrew W. Stephens, Marianne Patt, Andreas Schildan, Gusalija Behnisch, Constanze I. Seidenbecher, Björn H. Schott, Hermann Esselmann, Jens Wiltfang, Henryk Barthel, Osama Sabri, Michael C. Kreißl, Emrah Düzel, Anne Maass

**Affiliations:** ^1^ Institute of Cognitive Neurology and Dementia Research (IKND), Otto‐von‐Guericke University, Magdeburg Germany; ^2^ German Center for Neurodegenerative Diseases (DZNE), Magdeburg Germany; ^3^ Department for Psychiatry and Psychotherapy, University Medical Center Göttingen (UMG), Göttingen Germany; ^4^ Department of Nuclear Medicine, University of Leipzig, Leipzig Germany; ^5^ Life Molecular Imaging GmbH, Berlin Germany; ^6^ Clinic for Nuclear Medicine, University Hospital Augsburg, Augsburg Germany; ^7^ Leibniz Institute for Neurobiology (LIN), Magdeburg Germany; ^8^ German Center for Neurodegenerative Diseases (DZNE), Goettingen Germany; ^9^ University Medical Center Goettingen (UMG), Goettingen Germany; ^10^ Department of Psychiatry and Psychotherapy, University Medical Center, University of Goettingen, Goettingen Germany; ^11^ Otto von Guericke University, Magdeburg Germany

## Abstract

**Background:**

While some memory decline in old age is “normal”, there are some older individuals with maintained high cognitive performance. Using a multimodal approach including neuroimaging, fitness, genetic and questionnaire data (Fig1A), we aimed to identify factors that are related to successful cognitive aging and whether these differ between sexes.

**Method:**

We analyzed 165 cognitively normal older adults age = 60 years from an ongoing study (SFB1436) (age = 71±8years, 43% female). For all participants, we determined plasma Abeta1‐42/Abeta1‐40. Temporal lobe tau burden was estimated by [18F]PI‐2620 in a subsample (see Fig.1A for sample sizes). We assessed global white matter hyperintensity (WMH) volumes and gray matter thickness for medial temporal lobe (MTL), anterior cingulate cortex (ACC) and whole brain. We measured aerobic and muscular capacity (and blood pressure) by fitness assessment and trait/state anxiety by self‐reports. Genetic profiling included KLOTHO and KIBRA polymorphisms and APOE genotype. To phenotype successful cognitive aging, we i) grouped individuals age = 79.5 years into SuperAgers (N = 18) based on delayed verbal recall performance = normative values at age of 50‐60 years versus typical agers (N = 19). For the whole sample we ii) calculated cognitive age gap (CAG) as the difference between cognition‐predicted age and chronological age (Fig.3A). We assessed how markers of pathology, brain structure, fitness, mental health and genetics were related to CAG, covarying for chronological age, sex and education.

**Result:**

SuperAgers and typical agers did not differ in age, sex, education, fitness, anxiety or Abeta42/40 (all p‐values>0.1). However, SuperAgers had less WMH volume, higher ACC thickness, lower blood pressure and less temporal lobe tau‐tracer binding (small subgroup;). In the whole sample, younger cognitive age related to higher MTL and global cortical thickness, less temporal tau‐tracer binding, less anxiety (all p<0.05; Figure 3B) and marginally to higher muscular capacity (p = .06). Only the association between anxiety measures and CAG was moderated by sex (Fig.3B). CAG was not related to genotype.

**Conclusion:**

Our results suggest that successful cognitive aging is related to resistance against age‐related pathology and higher brain integrity. Younger cognitive age is linked to better mental health, especially in females.